# Supplementary material for: The genomic landscape of Vk*MYC myeloma highlights shared pathways of transformation between mice and humans
Source: Nat Commun. 2024 May 7;15:3844. doi: 10.1038/s41467-024-48091-w (PMC11076575; doi:10.1038/s41467-024-48091-w)

SUPPLEMENTARY INFORMATION

The genomic landscape of Vk*MYC myeloma highlights shared pathways of transformation between mice and humans.

Francesco Maura[1*], David G. Coffey[1*], Caleb K. Stein[2], Esteban Braggio[2], Bachisio Ziccheddu[1], Meaghen E. Sharik[2], Megan T. Du[2], Yuliza Tafoya Alvarado[2], Chang-Xin Shi[2], Yuan Xiao Zhu[2], Erin W. Meermeier[2], Gareth J. Morgan[3], Ola Landgren[1], P. Leif Bergsagel[2†], Marta Chesi[2†]

Supplementary Figure 1. Overview of methods.
Magnetically purified tumor DNA was analyzed from 96 Vκ*MYC mice which included 37 *de novo* mice that developed clonal plasma cell expansion, 38 recipient mice transplanted with tumor cells from *de novo* donors, and 20 *in vitro* cultured tumor cells derived from *de novo* or transplanted mice (**Figure 1, Supplemental Table 1**). DNA was analyzed by whole genome sequencing (WGS), whole exome sequencing (WES), mate pair whole genome sequencing, and array comparative genomic hybridization (aCGH) for single nucleotide variants (SNVs), copy number alterations (CNAs), and structural variants (SV). Tumor cell gene expression (GE) was analyzed by RNA sequencing in 91 Vκ*MYC MM. This figure was generated using Biorender.

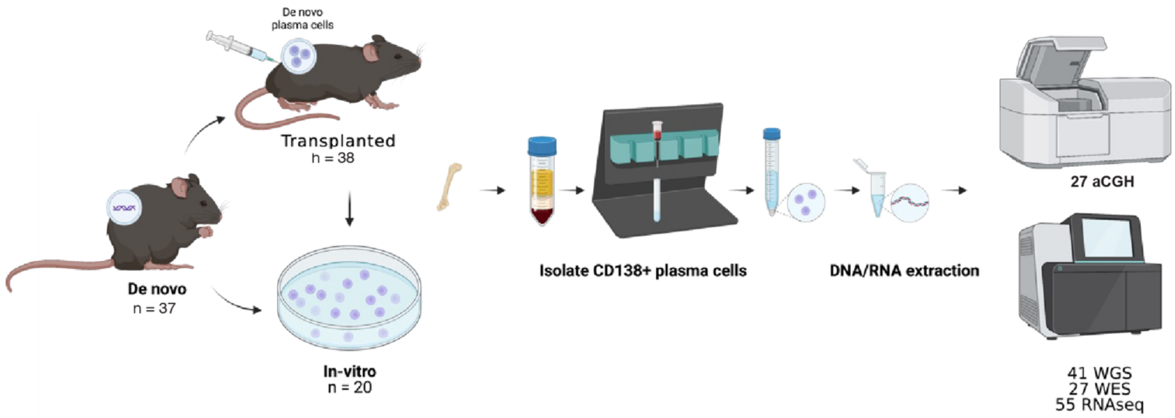

Supplementary Figure 2. Vk*MYC multiple myeloma (MM) mutational landscape. **A)** Similar mutational burden between WGS (n=41) and WES (n=27) Vk*MYC MM. **B)** Driver genes extracted by *dndscv* with significant q-value (i.e. <0.01). **C)** Patterns of co-occurrence and mutually exclusivity between mutations in driver genes. **D)** VAF of all nonsynonymous driver mutations. **E)** Distribution of non-coding and coding mutations across *Pten* first exons in samples with mutations, **F)** Distribution of non-coding and coding mutations across *Dusp2* in all unique tumors analyzed by WGS.

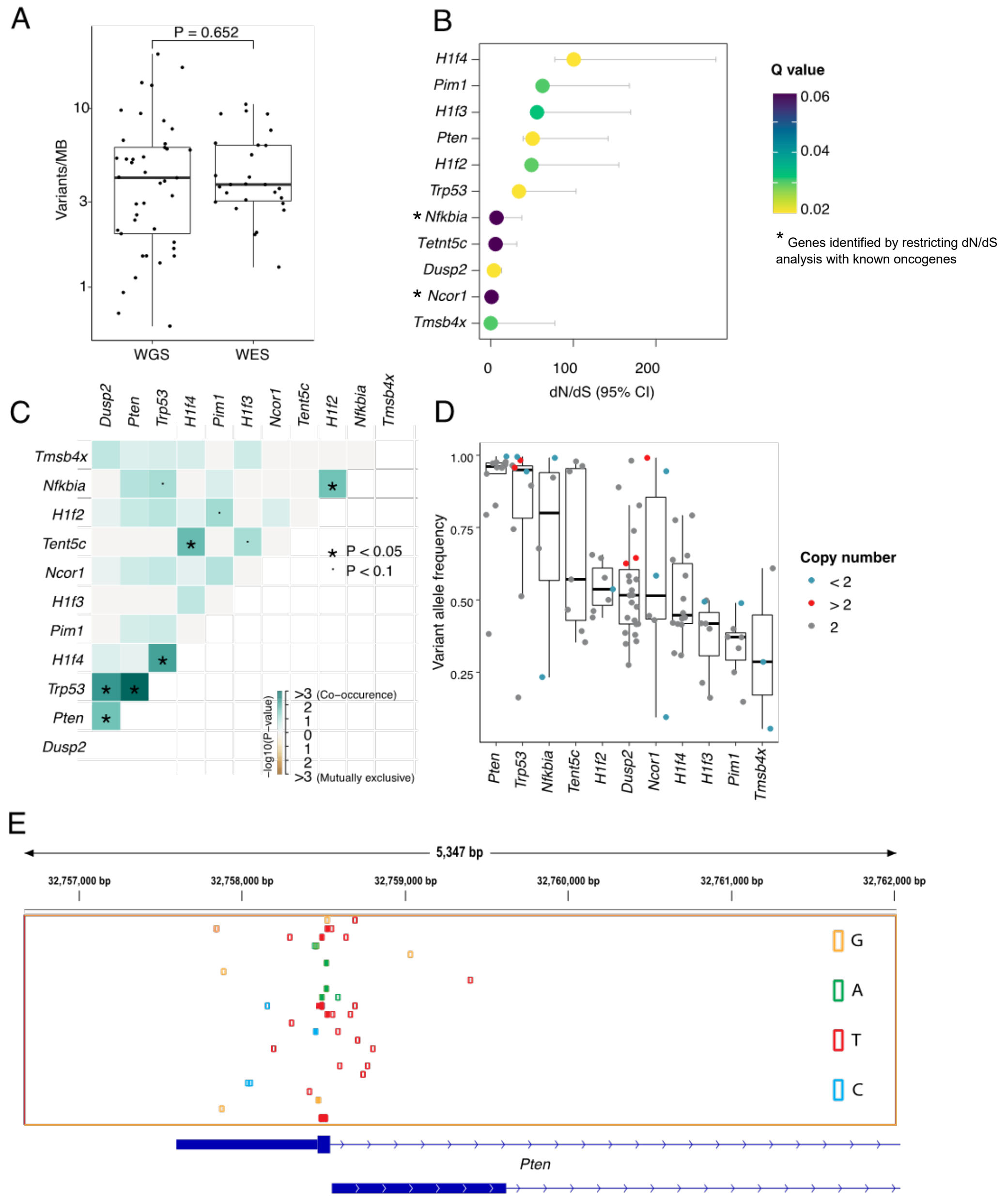

F

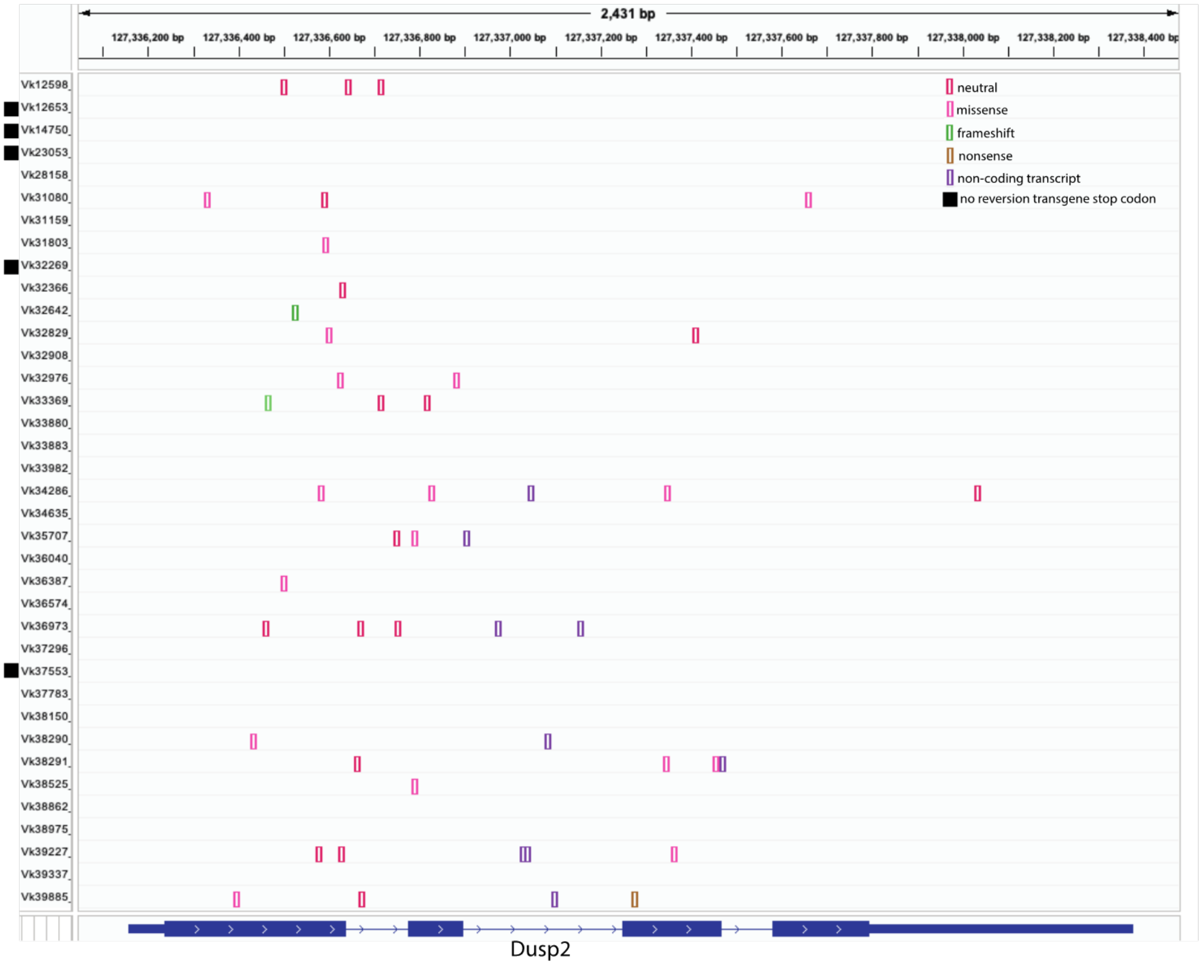

2,431 bp
127,336,200 bp
127,336,400 bp
127,336,600 bp
127,336,800 bp
127,337,000 bp
127,337,200 bp
127,337,400 bp
127,337,600 bp
127,337,800 bp
127,338,000 bp
127,338,200 bp
127,338,400 bp
Vk12598
Vk12653
Vk14750
Vk23053
Vk28158
Vk31080
Vk31159
Vk31803
Vk32269
Vk32366
Vk32642
Vk32829
Vk32908
Vk32976
Vk33369
Vk33880
Vk33883
Vk33982
Vk34286
Vk34635
Vk35707
Vk36040
Vk36387
Vk36574
Vk36973
Vk37296
Vk37553
Vk37783
Vk38150
Vk38290
Vk38291
Vk38525
Vk38862
Vk38975
Vk39227
Vk39337
Vk39885
neutral
missense
frameshift
nonsense
non-coding transcript
no reversion transgene stop codon
Dusp2

Supplementary Figure 3. Recurrent copy number alterations involving focal GISTIC peaks in Vk*MYC MM tumors (n=68).

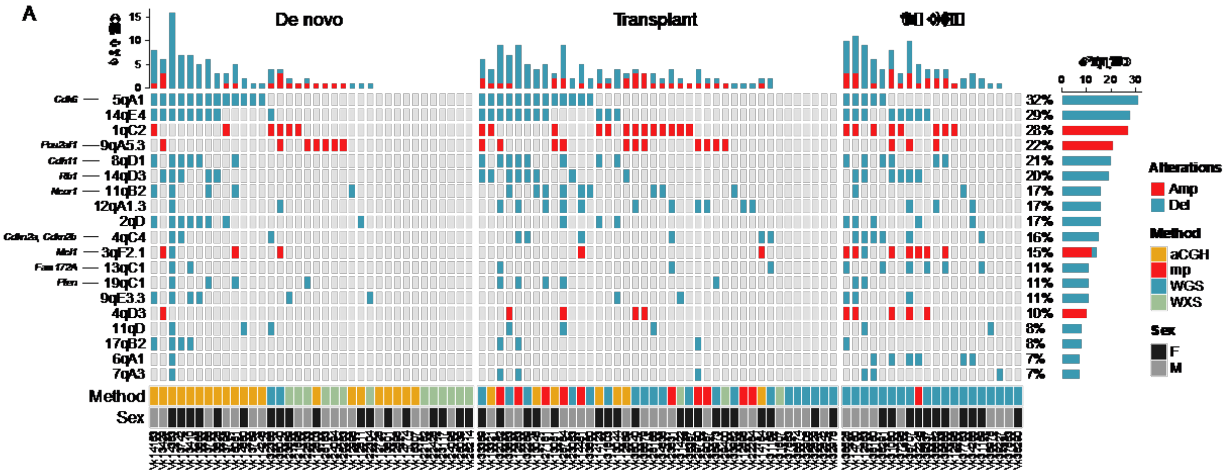

Supplementary Figure 4. Syntenic map of autosomal chromosomes
Murine (outer circle) and human (inner circle) (https://syntenybrowser.jax.org/browser).

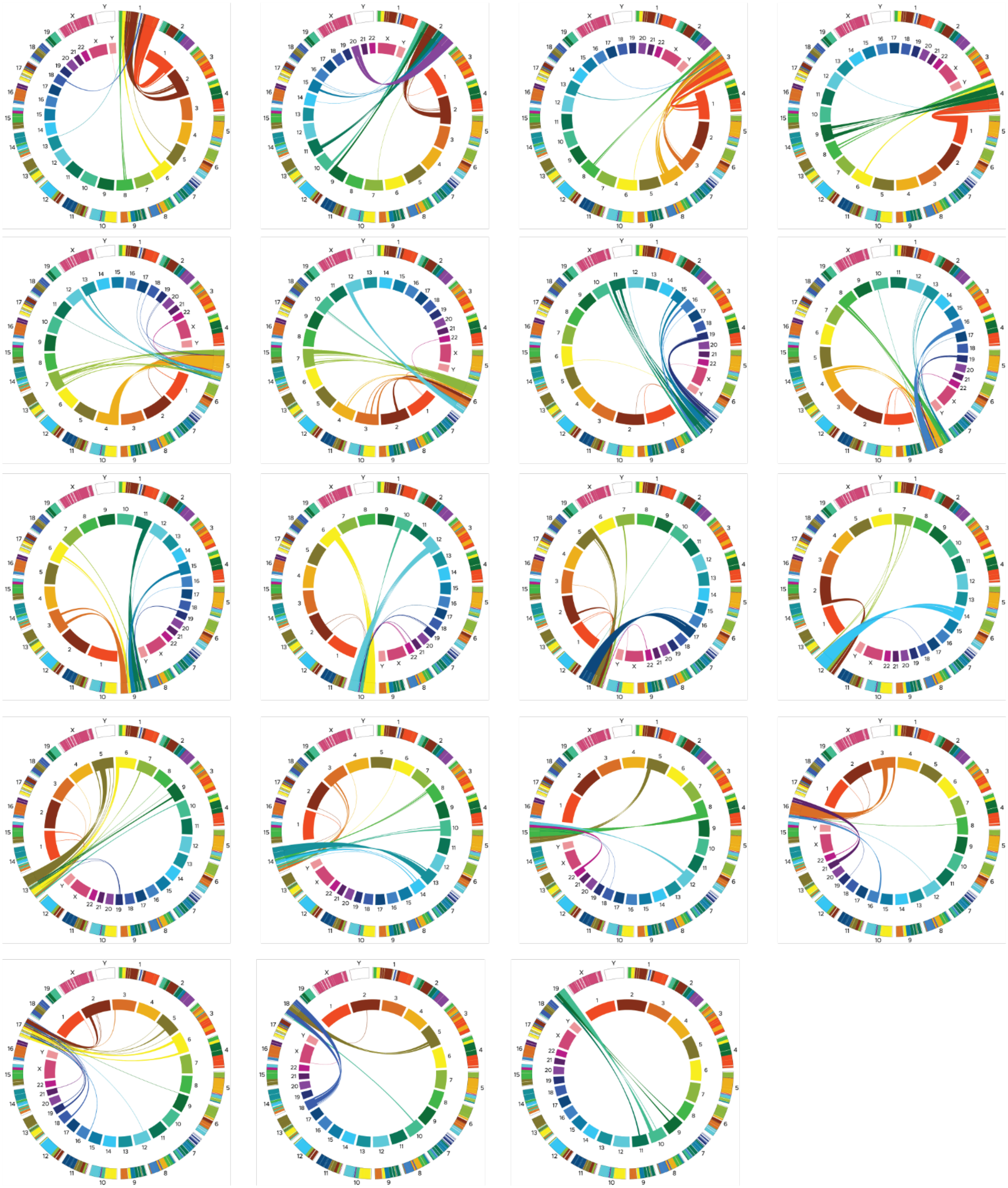

Supplementary Figure 5. Heat map summarizing copy number abnormalities in different human MM subgroups (n=673).

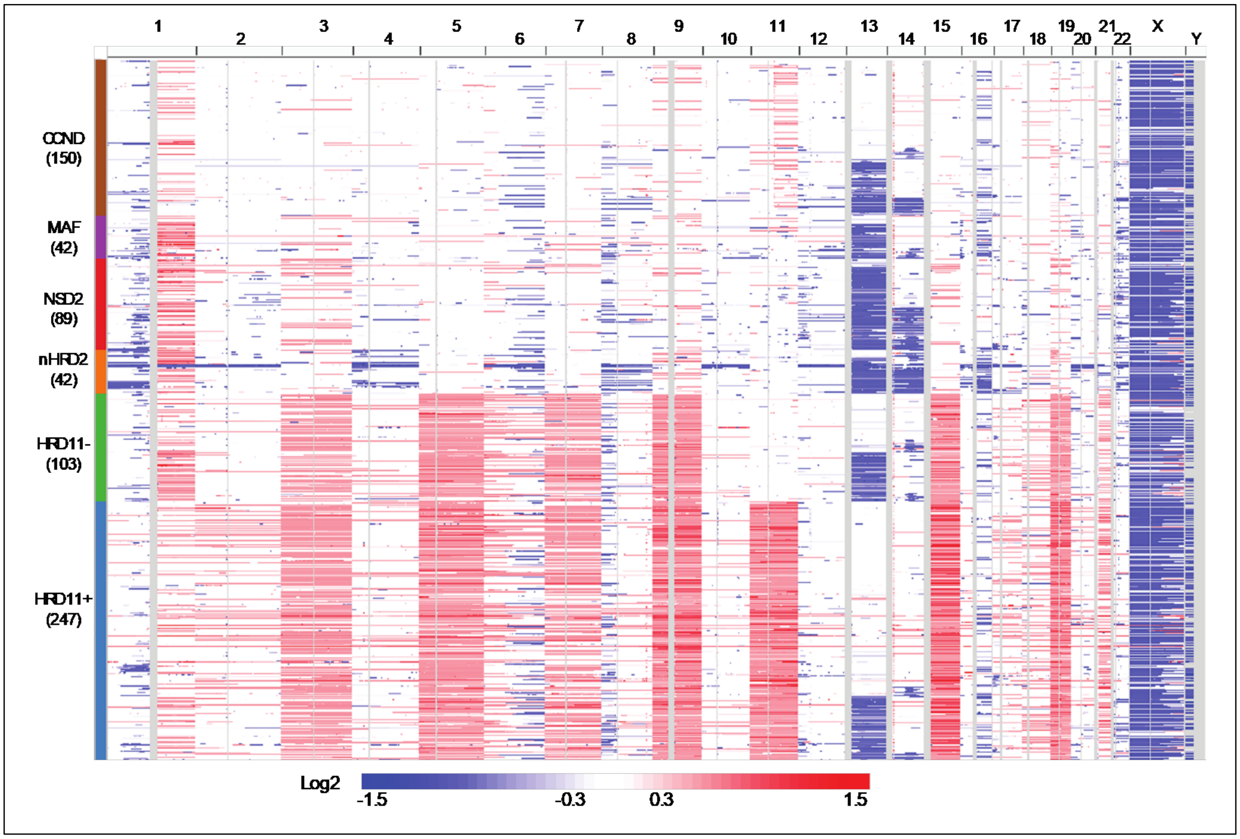

Supplementary Figure 6. Tumor evolution in serial samples at different stages of MM progression. A) Heat map of copy number abnormalities across the genome, **B)** at the IgH locus, **C)** or at *Kdm6a*, Numbers in B) indicate the log2 values for the deleted area.

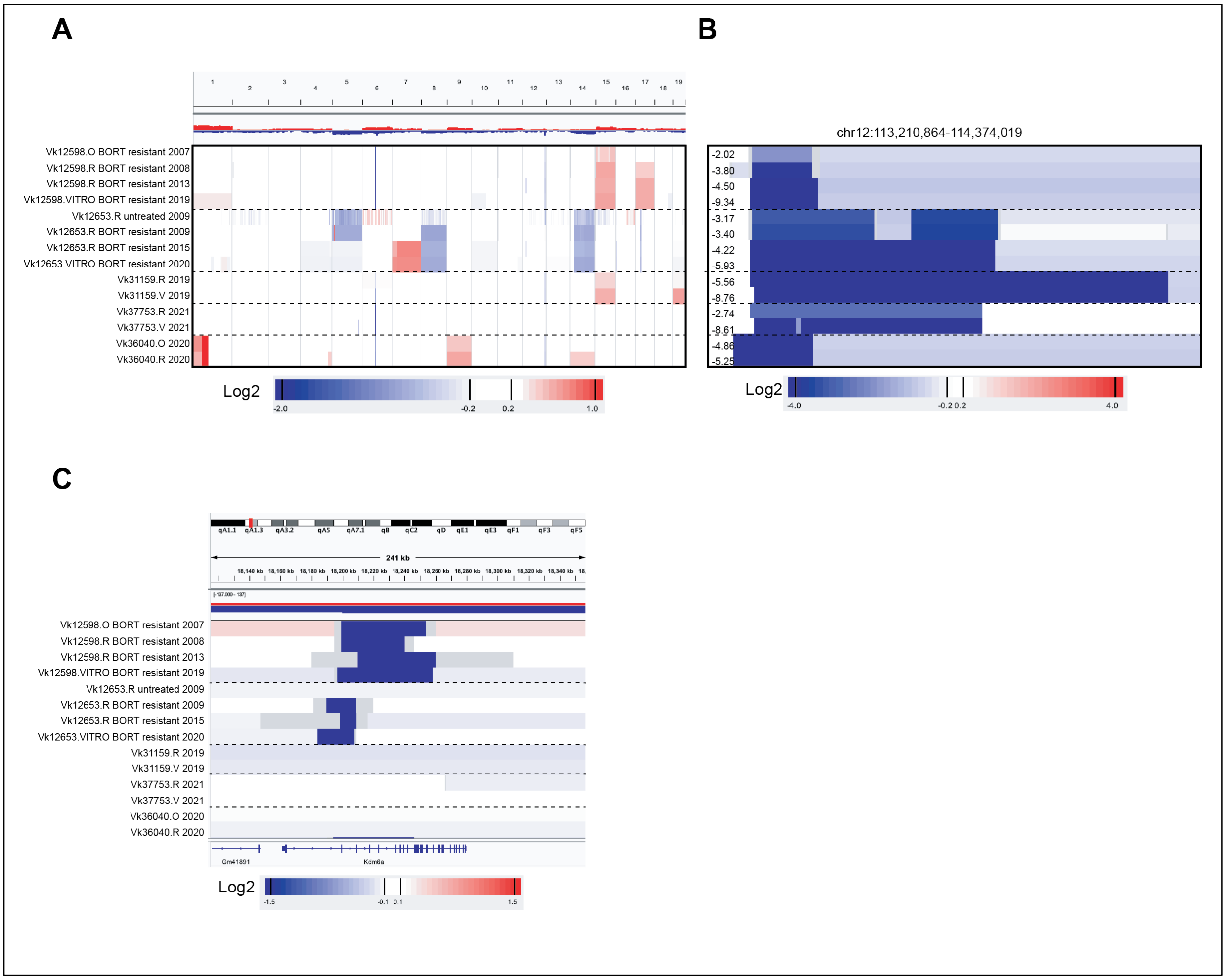

Supplementary Figure 7. Molecular time (0 equals early, 1 equals late) of 34 large gains in 19 Vk*MYC MM. Confidence of interval were generated using the molecular time bootstrap function (https://github.com/UM-Myeloma-Genomics).

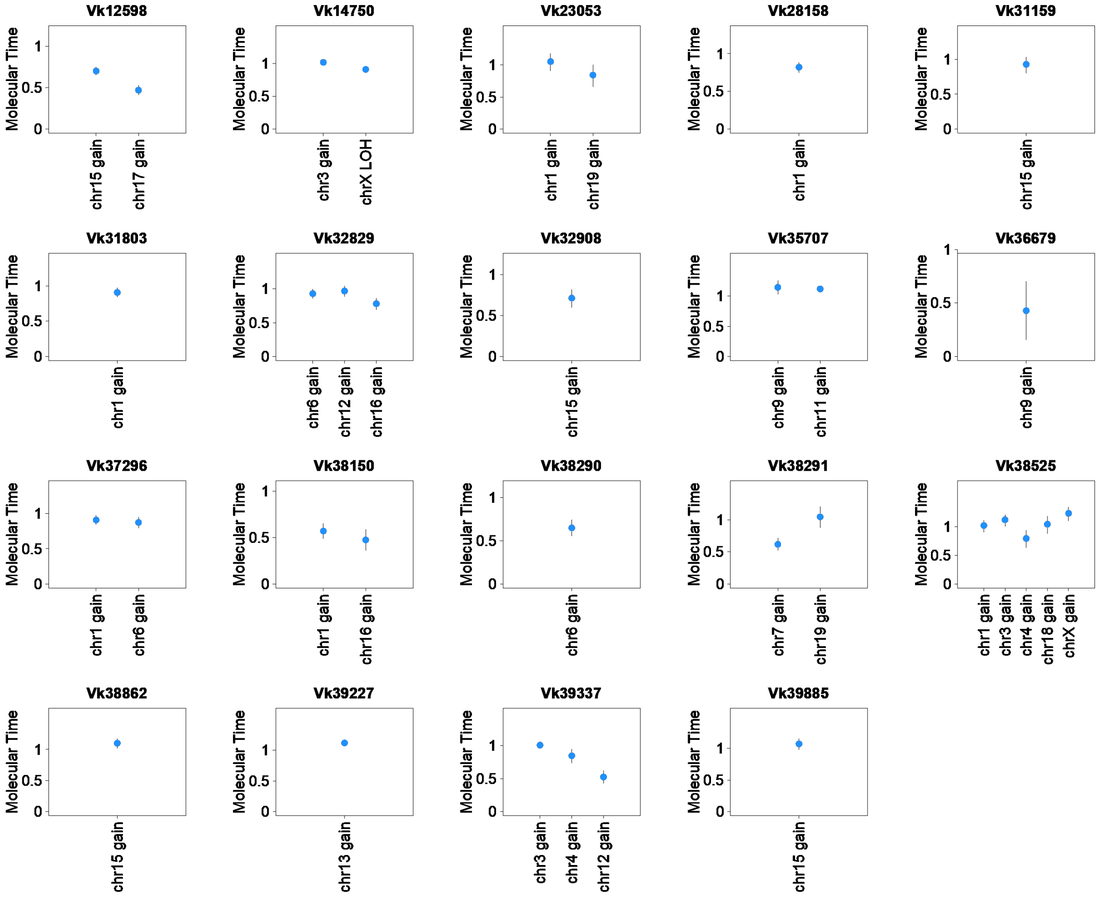

Supplementary Figure 8. NFKB index by stage in murine and human MM

A For murine MM NFkB Index increases from *de novo* to transplant to *in vitro* (Jonckheere-Terpstra test of ordered differences yields a p-value < 0.05).

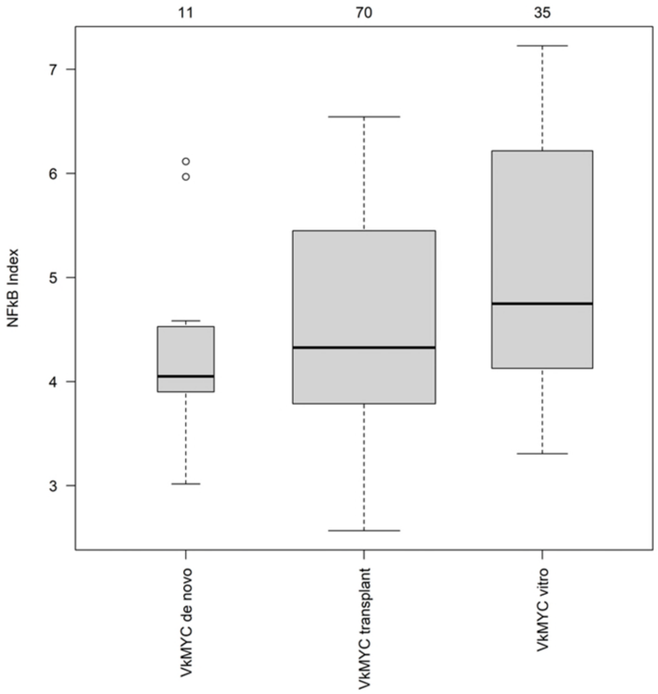

B For human MM NFkB Index increases from newly diagnosed to relapse (two-sided t-test p-value < 1e-13).

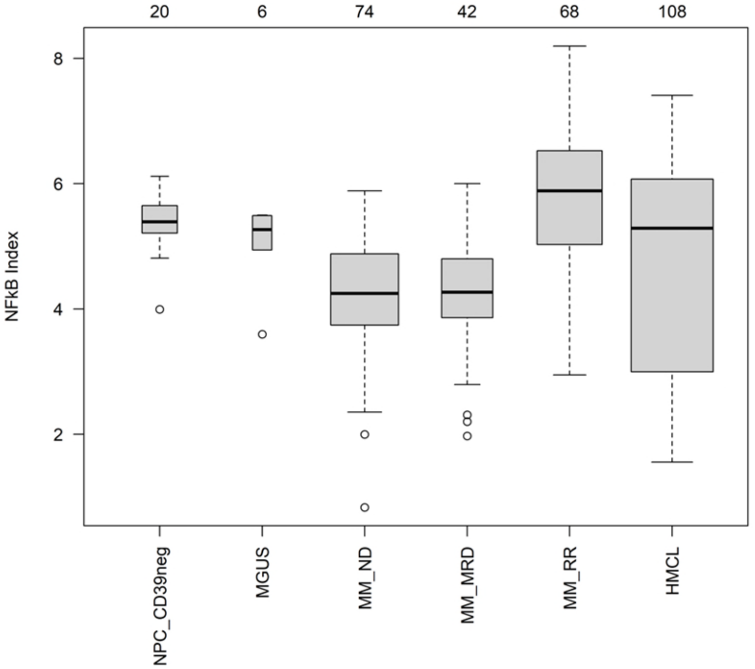

Supplementary Figure 9. Genetic deletion of the super-enhancer induces MM response.

A) Graphic representation of the Vk*MYC construct (not to scale), with head to tail integration. Green squares represent the kappa variable region and human MYC exons. The position of the two LoxP sites flanking the 3' Kappa enhancer is shown. Horizontal arrow indicates the transcription start point. Arrowhead at the bottom show the position and orientation of PCR primers. **B)** Detection of floxed versus unfloxed Vk*MYC allele by competitive qPCR using primers 1727, 1728 and 1875, shown in A), performed on spleen and BM harvested from Vk22284 tumor bearing mice at the indicated time before and after tamoxifen treatment. **C)** The relative abundance of floxed versus unfloxed Vk*MYC alleles after tamoxifen treatment, as shown in B), determined by qPCR using the Δct method. Bars represent mean with standard deviation. **D)** M-spikes over time in four Vk22284 tumor bearing mice treated with 1 mg tamoxifen by i.p injection on days 1-5 and 36-40 (yellow shading). **E)** Detection of floxed and unfloxed Vk*MYC allele by competitive PCR as in B), performed on tumor DNA extracted at necropsy from two relapsing Vk22284 tumor bearing mice 11 weeks after receiving tamoxifen, indicating that tumor cells with incomplete floxing of the Vk*MYC allele are likely selected over time and drive tumor relapse.

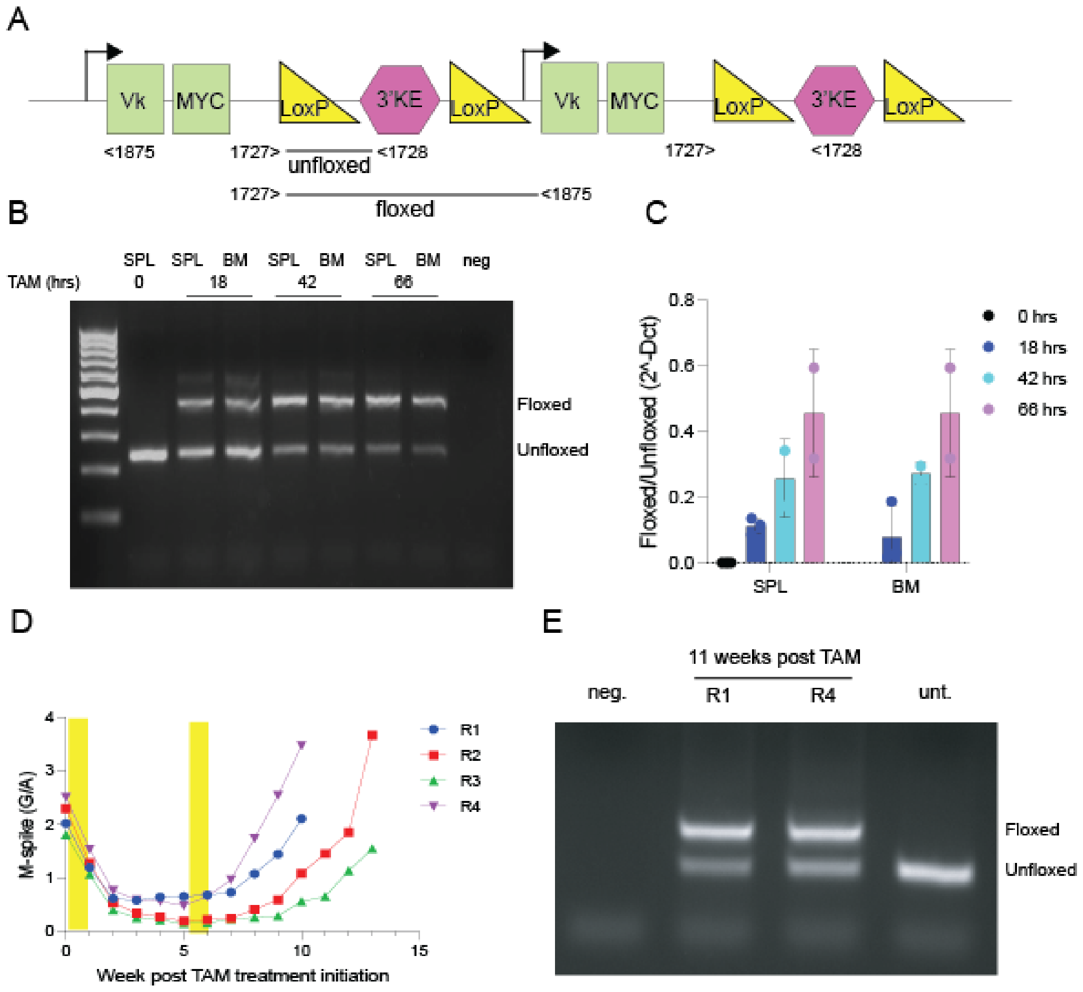

Supplementary Figure 10. AID and somatic hypermutation activity in Vk*MYC (n=41) and human multiple myeloma (n=30). SBS= single base substitutions.

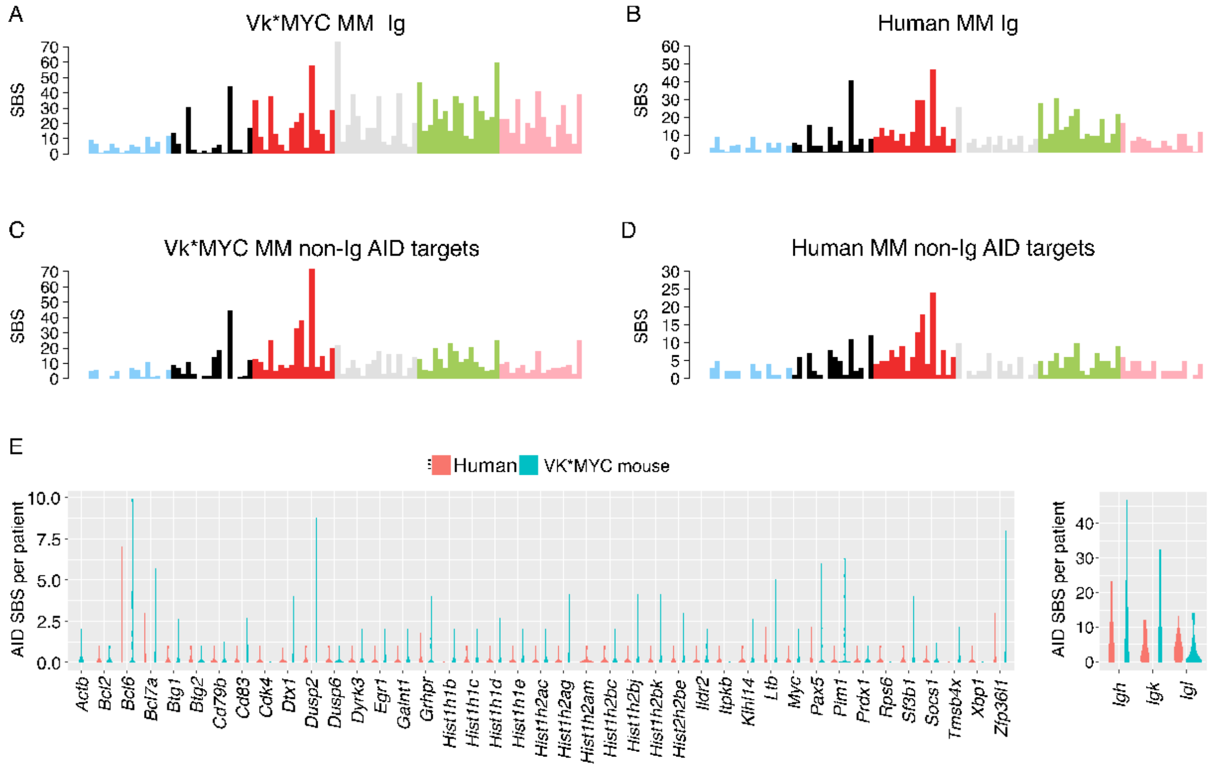

Supplementary Figure 11. APOBEC mutational contribution and expression in whole exome sequencing (A) and RNAseq data (B) from Vk*MYC multiple myeloma.

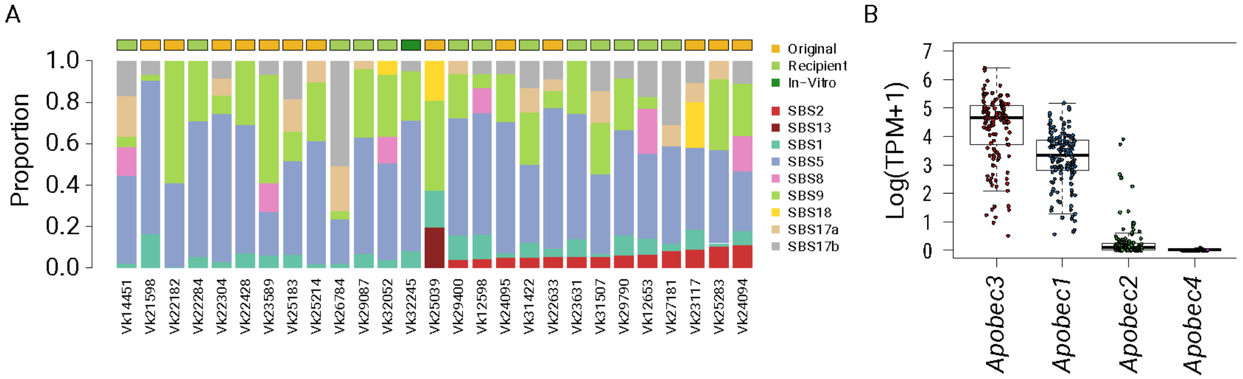

Supplementary Figure 12. scRNA of 15 Vk*MYC multiple myeloma.

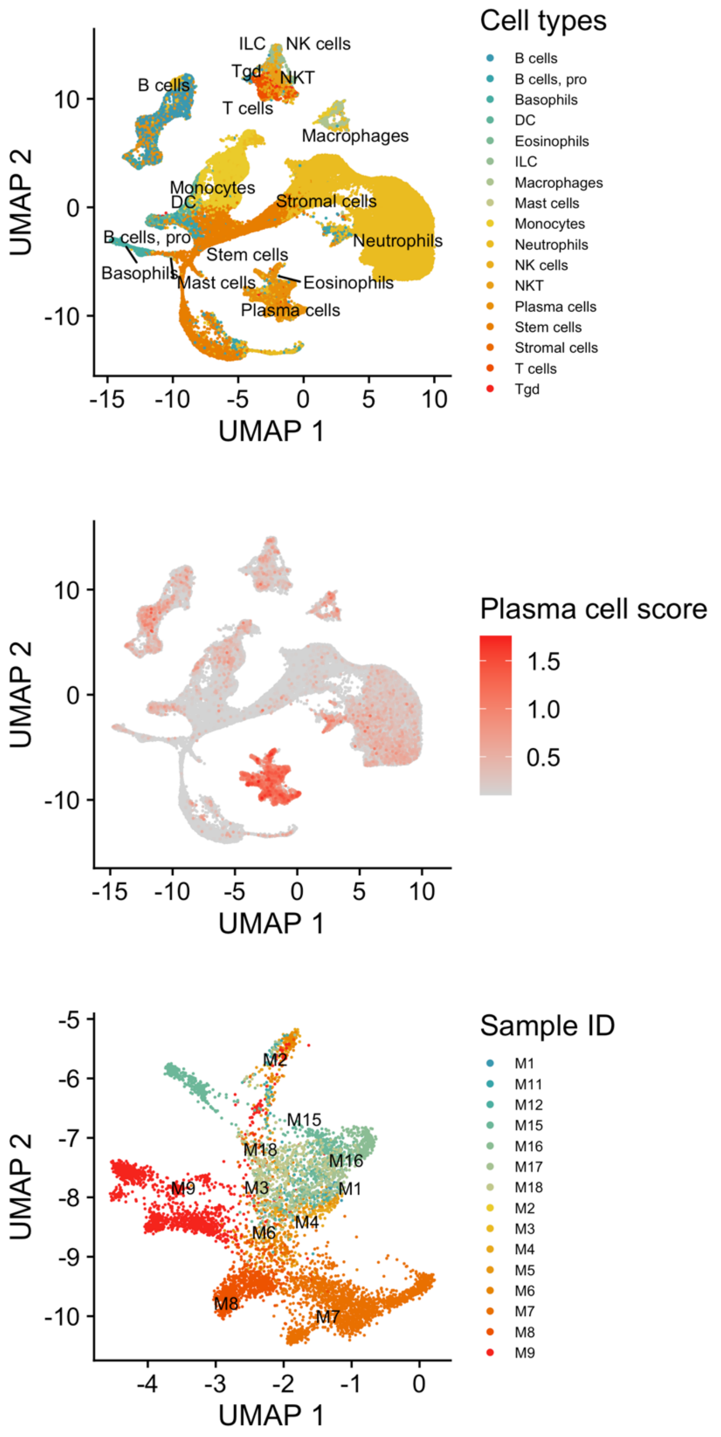

Supplementary Figure 13. Mutational signatures differential contribution across different stages. No statistically significant differences were observed using pairwise.wilcoxon test.

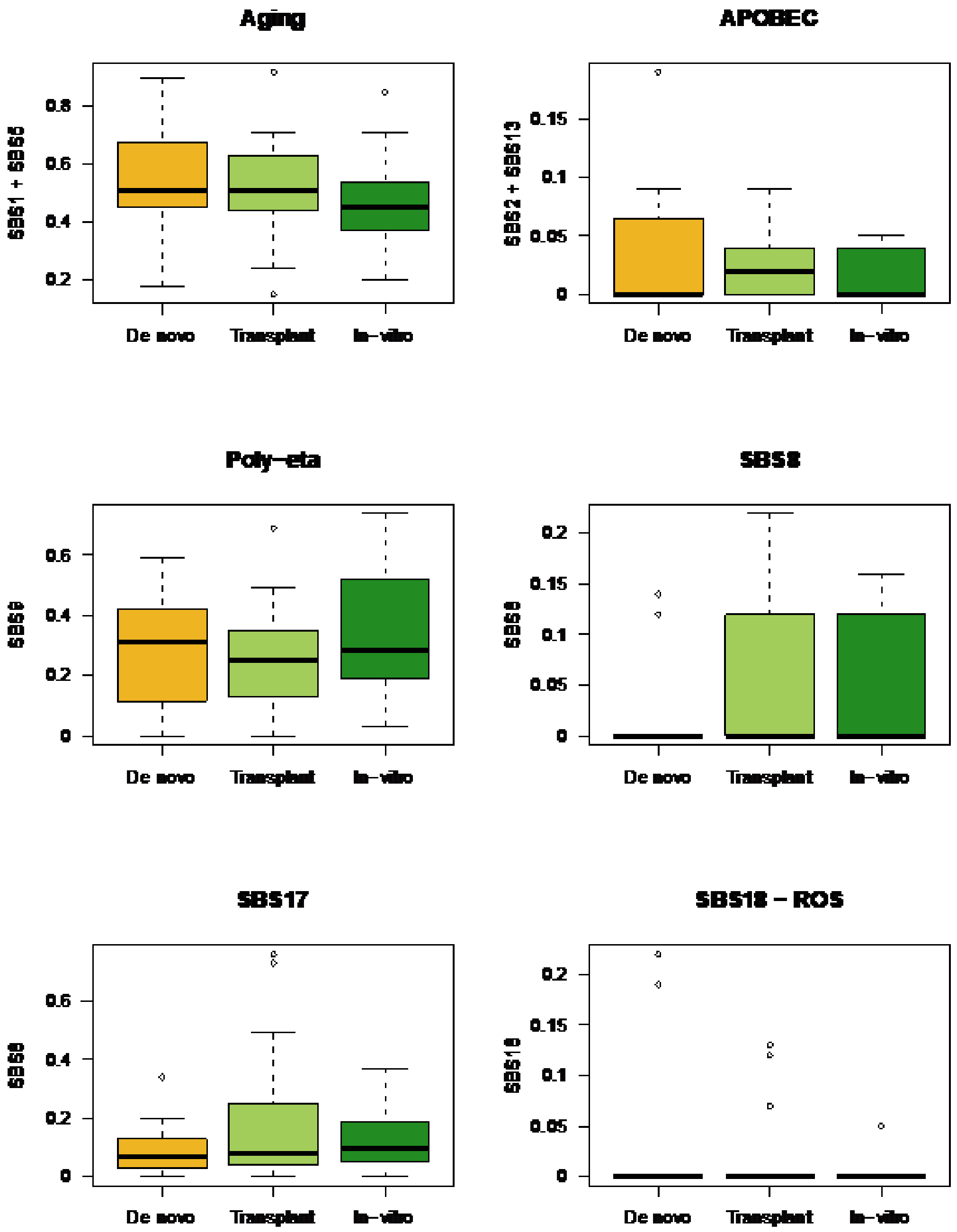

Supplement: Supplementary file 1 — Supplementary Information [file 41467_2024_48091_MOESM1_ESM.pdf]
