# Supplementary material for: The genomic landscape of Vk*MYC myeloma highlights shared pathways of transformation between mice and humans
Source: Nat Commun. 2024 May 7;15:3844. doi: 10.1038/s41467-024-48091-w (PMC11076575; doi:10.1038/s41467-024-48091-w)
Supplement: Supplementary file 2 — Description of Additional Supplementary Files [file 41467_2024_48091_MOESM2_ESM.pdf]

Description of Additional Supplementary Files

Supplementary Data 1

Description: Characteristics of VK*MYC MM.

Supplementary Data 2

Description: Sequencing coverage of 178 samples from 96 multiple myeloma from VkMYC mice, and 3 tails from BioProject PRJNA938752.

Supplementary Data 3

Description: Summary of mice strains.

Supplementary Data 4

Description: List of all nonsynonymous mutations across 81 VkMYC MM interrogated with either WES or WGS.

Supplementary Data 5

Description: Mutation burden pairwise Wilcoxon test P values.

Supplementary Data 6

Description: Somatic nucleotide variants pairwise Fisher test P values.

Supplementary Data 7

Description: CNV calls from 96 Vk*MYC MM.

Supplementary Data 8

Description: GISTIC2.0 significant copy number aberrations.

Supplementary Data 9

Description: GISTIC2.0 significant whole chromosome events.

Supplementary Data 10

Description: Percentage (and number) of Vk*MYC MM tumors from different stages of progression with large chromosome gain and losses.

Supplementary Data 11

Description: Copy number variants pairwise Fisher test P values.

Supplementary Data 12

Description: Molecular time-based time windows for large chromosomal gains acquired in the same cases.

Supplementary Data 13

Description: Molecular time of 34 large gains across 19 Vk*MYC mouse MM.

Supplementary Data 14

Description: Catalog of SV in 52 Vk*MYC MM.

Supplementary Data 15

Description: Comparison of SV events between different stages.

Supplementary Data 16

Description: Mapping of IgH translocations within the IgH locus Vk*MYC MM tumors.

Supplementary Data 17

Description: Samples with outlier expression and IAP LTR insertions of Map3k14, Il6, Ncor1 and Ltbr.

Supplementary Data 18

Description: Genetic and phenotypic features of available Vk*MYC transplantable lines. Expression (TPM) of drug targets Tnfrsf17 (BCMA) and Bcl2 is also reported.

Supplementary Data 19

Description: Genetic and phenotypic features of 25 Vk*MYC VITRO lines. Expression (TPM) of drug targets Tnfrsf17 (BCMA) and Bcl2 is also reported.

Supplementary Data 20

Description: Mutational signatures in VkMYC MM.

Supplementary Data 21

Description: Frequency (percentage) of common genomic abnormalities across different MM subtypes and Vk*MYC tumors.
