# Supplementary material for: The genomic landscape of Vk*MYC myeloma highlights shared pathways of transformation between mice and humans
Source: Nat Commun. 2024 May 7;15:3844. doi: 10.1038/s41467-024-48091-w (PMC11076575; doi:10.1038/s41467-024-48091-w)
Supplement: Supplementary file 5 — Reporting Summary [file 41467_2024_48091_MOESM5_ESM.pdf]

nature portfolio

Corresponding author(s): Marta Chesi

Last updated by author(s): Mar 17, 2024

Reporting Summary

Nature Portfolio wishes to improve the reproducibility of the work that we publish. This form provides structure for consistency and transparency in reporting. For further information on Nature Portfolio policies, see our Editorial Policies and the Editorial Policy Checklist.

Statistics

For all statistical analyses, confirm that the following items are present in the figure legend, table legend, main text, or Methods section.

n/a	Confirmed	
☐	☒	The exact sample size (*n*) for each experimental group/condition, given as a discrete number and unit of measurement
☐	☒	A statement on whether measurements were taken from distinct samples or whether the same sample was measured repeatedly
☐	☒	The statistical test(s) used AND whether they are one- or two-sided<br>*Only common tests should be described solely by name; describe more complex techniques in the Methods section.*
☐	☒	A description of all covariates tested
☐	☒	A description of any assumptions or corrections, such as tests of normality and adjustment for multiple comparisons
☐	☒	A full description of the statistical parameters including central tendency (e.g. means) or other basic estimates (e.g. regression coefficient) AND variation (e.g. standard deviation) or associated estimates of uncertainty (e.g. confidence intervals)
☐	☒	For null hypothesis testing, the test statistic (e.g. *F*, *t*, *r*) with confidence intervals, effect sizes, degrees of freedom and *P* value noted<br>*Give P values as exact values whenever suitable.*
☒	☐	For Bayesian analysis, information on the choice of priors and Markov chain Monte Carlo settings
☐	☒	For hierarchical and complex designs, identification of the appropriate level for tests and full reporting of outcomes
☐	☒	Estimates of effect sizes (e.g. Cohen's *d*, Pearson's *r*), indicating how they were calculated

Our web collection on statistics for biologists contains articles on many of the points above.

Software and code

Policy information about availability of computer code

Data collection

In this study we interrogated the genomic and/or transcriptomic landscape of 128 Vk*MYC MM samples representing 118 unique tumors from 39 de novo MM, 63 transplantable lines and 25 tumors capable of growing in vitro, to capture the mutations spontaneously selected during disease progression in an unbiased way.
In total we analyzed:
- 41 Whole genome sequencing (WGS)
- 27 Whole exome sequencing
- 28 aCGH
- 11 mate-pair WGS
- 89 RNAseq bulk
- 15 scRNA from publicly available data set: GSE15907, GSE37448

For comparisons between human and murine gene expression, CoMMpass IA19 release was used--limited to bone marrow samples with available RNA-Seq data and either a ‘baseline’ or ‘confirm progression’ reason for visit (phs000748.v3.p2).

Data analysis	Whole genomic and whole exome sequencing reads were aligned the mouse reference genome (GRCm38/mm10) using BWA.<br>Mutect2 was used to call mutations<br>CNV were called using the GATK4 Somatic CNV pipeline modified for mouse<br>SV were called using Lumpy (57) as implemented in Smoove release 19 (https://github.com/brentp/smoove)<br>Mutational signatures were estimated using first sigprofiler and hdp as de novo extraction, and then mmsig as fitting<br>Molecular time analysis was run as previously described (https://github.com/UM-Myeloma-Genomics/mol_time)

For manuscripts utilizing custom algorithms or software that are central to the research but not yet described in published literature, software must be made available to editors and reviewers. We strongly encourage code deposition in a community repository (e.g. GitHub). See the Nature Portfolio guidelines for submitting code & software for further information.

Data

Policy information about availability of data

All manuscripts must include a data availability statement. This statement should provide the following information, where applicable:

- Accession codes, unique identifiers, or web links for publicly available datasets
- A description of any restrictions on data availability
- For clinical datasets or third party data, please ensure that the statement adheres to our policy

WGS, Mate pair, WES and RNAseq datasets are available on NCBI Gene Expression Omnibus; RRID:SCR_005012; GSE255233 and NCBI Sequence Read Archive; RRID:SCR_004891; BioProject PRJNA938752 . Publicly available data from previous mouse models were imported and reanalyzed following the pipeline described above: BioProjects PRJNA560057, PRJNA721176, PRJNA910238, PRJNA259862, PRJNA845532, PRJNA846769, PRJNA881497, PRJNA912227 and PRJNA759563.

Research involving human participants, their data, or biological material

Policy information about studies with human participants or human data. See also policy information about sex, gender (identity/presentation), and sexual orientation and race, ethnicity and racism.

Reporting on sex and gender	All sex data from human data have been previously published and available thought MMRF research portal.
Reporting on race, ethnicity, or other socially relevant groupings	All relevant demographic data from human data have been previously published and available thought MMRF research portal.
Population characteristics	Human data have been used only to compare human myeloma with VkMYC myeloma. All the human data were already published and publicly available.
Recruitment	NA
Ethics oversight	NA

Note that full information on the approval of the study protocol must also be provided in the manuscript.

Field-specific reporting

Please select the one below that is the best fit for your research. If you are not sure, read the appropriate sections before making your selection.

☒ Life sciences ☐ Behavioural & social sciences ☐ Ecological, evolutionary & environmental sciences

For a reference copy of the document with all sections, see nature.com/documents/nr-reporting-summary-flat.pdf

Life sciences study design

All studies must disclose on these points even when the disclosure is negative.

Sample size	118 genetically engineered Vk*MYC mice with multiple myeloma. Sample size was not statistically pre-determined, but dictated by the number of samples available for the analysis
Data exclusions	Data were excluded only based on sequencing quality.
Replication	For genomics studies, we did not use biologic replicates but instead analyzed multiple independent tumors. For supplementary figure 9, we analyzed two independent tumors for each of the three time points, and qPCR analysis was performed in triplicate.
Randomization	Genomics analysis was performed on all available tumors, where randomization is not applicable. The treatment with tamoxifen (Supl. Figure 9) was done on mice selected for the appropriate genotype. No treatment randomization was performed.
Blinding	As no treatment randomization was performed in the study, no blinding was required. Group allocation for genomics analysis was determined only by disease status, and no randomization or blinding was applicable.

Reporting for specific materials, systems and methods

We require information from authors about some types of materials, experimental systems and methods used in many studies. Here, indicate whether each material, system or method listed is relevant to your study. If you are not sure if a list item applies to your research, read the appropriate section before selecting a response.

Materials & experimental systems

n/a	Involved in the study
☒	☐ Antibodies
☐	☒ Eukaryotic cell lines
☒	☐ Palaeontology and archaeology
☐	☒ Animals and other organisms
☒	☐ Clinical data
☒	☐ Dual use research of concern
☒	☐ Plants

Methods

n/a	Involved in the study
☒	☐ ChIP-seq
☒	☐ Flow cytometry
☒	☐ MRI-based neuroimaging

Eukaryotic cell lines

Policy information about cell lines and Sex and Gender in Research

Cell line source(s)	Generated in our laboratory from our mouse colony.
Authentication	Unique CDR3 sequence obtained by genomics analysis. Sequence provided in suppl. Table 19. Cellosaurus ID provided in Suppl. Table 1.
Mycoplasma contamination	All cell lines were tested for mycoplasma contamination biannually using the MycoAlert kit (Promega)
Commonly misidentified lines (See ICLAC register)	None

Animals and other research organisms

Policy information about studies involving animals; ARRIVE guidelines recommended for reporting animal research, and Sex and Gender in Research

Laboratory animals	VkMYC mouse models and derivative crosses. Mouse strains are listed in Suppl. Table 3; genotype, sex and age of each individual mouse used in the study are listed in Suppl. Table 1
Wild animals	No wild anymals were used in the study
Reporting on sex	Sex of each mouse is reported in Supplementary Table 1.
Field-collected samples	All samples were collected in Bergsagel and Chesi's lab at Mayo Clinic, AZ. No animals were collected from the field
Ethics oversight	All experiments were performed under the approval of the Mayo Foundation Institutional Animal Care and Use Committee (protocol A00001948-16-R22) and conformed to all the regulatory environmental safety standards

Note that full information on the approval of the study protocol must also be provided in the manuscript.
